# Characterisation of a Multi-ligand Binding Chemoreceptor CcmL (Tlp3) of *Campylobacter jejuni*

**DOI:** 10.1371/journal.ppat.1003822

**Published:** 2014-01-02

**Authors:** Hossinur Rahman, Rebecca M. King, Lucy K. Shewell, Evgeny A. Semchenko, Lauren E. Hartley-Tassell, Jennifer C. Wilson, Christopher J. Day, Victoria Korolik

**Affiliations:** Institute for Glycomics, Griffith University, Gold Coast Campus, Gold Coast, Australia; University of Illinois, United States of America

## Abstract

*Campylobacter jejuni* is the leading cause of human gastroenteritis worldwide with over 500 million cases annually. Chemotaxis and motility have been identified as important virulence factors associated with *C. jejuni* colonisation. Group A transducer-like proteins (Tlps) are responsible for sensing the external environment for bacterial movement to or away from a chemical gradient or stimulus. In this study, we have demonstrated Cj1564 (Tlp3) to be a multi-ligand binding chemoreceptor and report direct evidence supporting the involvement of Cj1564 (Tlp3) in the chemotaxis signalling pathway via small molecule arrays, surface plasmon and nuclear magnetic resonance (SPR and NMR) as well as chemotaxis assays of wild type and isogenic mutant strains. A modified nutrient depleted chemotaxis assay was further used to determine positive or negative chemotaxis with specific ligands. Here we demonstrate the ability of Cj1564 to interact with the chemoattractants isoleucine, purine, malic acid and fumaric acid and chemorepellents lysine, glucosamine, succinic acid, arginine and thiamine. An isogenic mutant of *cj1564* was shown to have altered phenotypic characteristics of *C. jejuni*, including loss of curvature in bacterial cell shape, reduced chemotactic motility and an increase in both autoagglutination and biofilm formation. We demonstrate Cj1564 to have a role in invasion as in *in vitro* assays the *tlp3* isogenic mutant has a reduced ability to adhere and invade a cultured epithelial cell line; interestingly however, colonisation ability of avian caeca appears to be unaltered. Additionally, protein-protein interaction studies revealed signal transduction initiation through the scaffolding proteins CheV and CheW in the chemotaxis sensory pathway. This is the first report characterising Cj1564 as a multi-ligand receptor for *C. jejuni*, we therefore, propose to name this receptor CcmL, *Campylobacter* chemoreceptor for multiple ligands. In conclusion, this study identifies a novel multifunctional role for the *C. jejuni* CcmL chemoreceptor and illustrates its involvement in the chemotaxis pathway and subsequent survival of this organism in the host.

## Author Summary

Bacterial chemotaxis is an important part in initiation of colonisation and subsequent pathogenicity. In this study, we report direct evidence supporting the involvement of *C. jejuni* transducer-like protein Cj1564 (Tlp3) in the chemotaxis signalling pathway via small molecule arrays, surface plasmon and nuclear magnetic resonance (SPR and NMR) as well as chemotaxis assays of wild type and isogenic mutants. We further demonstrate its ability to interact with chemoattractants isoleucine, purine, malic acid and fumaric acid and chemorepellents lysine, glucosamine, succinic acid, arginine and thiamine. This is the first report identifying Cj1564 as a multi-ligand receptor for *Campylobacter jejuni* and its signal transduction initiation through the CheV and CheW proteins. Finally, our characterisation of *C. jejuni* Cj1564 provides additional basis for elucidating the roles of other group A chemoreceptors and their importance in the chemotaxis signalling pathway.

## Introduction

*Campylobacter jejuni* is the one of the prevalent causes of acute human bacterial gastroenteritis worldwide [1]–[3]. *C. jejuni* is commonly found in the gastrointestinal tract of birds and poultry as commensal microbial flora [1], with infections in humans usually occurring from consumption of undercooked poultry, unpasteurised milk or untreated water. Symptoms include development of abdominal pains, fever and diarrhoea which can contain blood and leukocytes [4], [5]. Additionally *Campylobacter* enteritis is associated with post infectious complications ranging from reactive arthritis or reactive myositis to the more severe Guillain-Barré syndrome [1], [6]–[8].

The development of disease depends on the ability of bacteria to adapt to the environment of the human gut [9]. To date, it is known that factors associated with virulence and pathogenicity of *C. jejuni* include iron acquisition, chemotaxis, adherence and lipooligosaccharides (LOS) [5], [10], [11]. *C. jejuni* is known to be highly motile in viscous environments, with motility and swimming velocity increasing with increasing viscosity [12]. Chemotaxis, the ability of bacterial cells to detect temporal changes in the chemical concentration of their surrounding environment, and flagella-mediated motility, have been reported to play an important role in the intestinal colonisation of avian and mammalian hosts, as well as the invasion of intestinal epithelial cells [10], [12]–[17]. Furthermore, Hugdahl *et al.* (1988) have identified chemoattractants that *C. jejuni* is preferentially motile towards, which include amino acids found in the gastrointestinal tract, organic acid intermediates of the TCA cycle and components of mucous such as mucin. [18]. The importance of *C. jejuni* chemotaxis and motility in colonisation and pathogenicity has previously been shown with non-motile mutants defective in chemotaxis, unable to colonise and cause disease in the gastrointestinal tract of mice [13], [19]–[21]. Furthermore, strains with mutations in chemotactic genes and flagella associated genes were not capable of colonising chicken caeca [14] and lost the ability to colonise rabbits [22] and ferrets [20]. Another important observation was that mutants defective in chemotactic motility also lost the ability to autoagglutinate, adhere and invade mammalian cells [12], [15], [16], [23], [24].

The fundamental components of the chemotaxis signalling pathway, which are conserved in all motile prokaryotes, consist of the chemoreceptors, a cytoplasmic histidine kinase, CheA, a coupling or scaffolding protein, CheW and/or CheV and a response regulator, CheY. In the well-characterised chemotaxis system of *E. coli*, CheA is regulated by the chemoreceptors through association with CheW and uses ATP to autophosphorylate a specific histidine residue. The phosphoryl group is subsequently transferred to the response regulator CheY [25]. Phospho-CheY interacts with the flagella motor to induce clockwise (CW) rotation resulting in a tumbling motion, where the cell momentarily stops and randomly reorientates [25]–[28]. Attractant-bound chemoreceptors inhibit CheA kinase activity, with a resulting decrease in the levels of phospho-CheY. In contrast, repellent-bound or empty chemoreceptors stimulate CheA kinase activity, thereby increasing phospho-CheY levels and tumbling events, orientating the cell in a new direction [25], [26], [28], [29]. It is important to note that in *C. jejuni*, although the basic chemotaxis pathway backbone, consisting of Receptor-CheA-CheW(V)-CheY, is conserved, there are a number of differences to the paradigm *E. coli* model. *C. jejuni* encodes a two-domain CheA protein that includes a CheY-like response regulator domain in addition to the traditional histidine kinase domain, and a two domain CheV protein consisting of a CheW-like scaffolding domain and a CheY-like response regulator domain, as well as the paradigm CheW protein. *C. jejuni* also has a unique CheB protein that lacks a CheY-like response regulator domain found in all other bacterial chemotaxis pathways characterised to date [30].

Ten chemoreceptors have been identified in *C. jejuni* with homology to the methyl-accepting chemotaxis proteins (MCPs) in *E. coli* and have been designated Transducer-like proteins, Tlps. Group A Tlp receptors include CcaA (Tlp1), Tlps 2, 3, 4, 7 and 10 which have similarities to *E. coli* MCP structures and family A transducers of *Halobacterium salinarium* [30], [31]. Group A Tlps are thought to sense extracellular ligands and consist of a periplasmic sensory domain which is variable between different receptors, two transmembrane domains, and the highly conserved C-terminal cytoplasmic signalling domain [30], [32]. The sensory domain of each Tlp appears to be unique from that of non-Epsilon proteobacterial chemoreceptors; therefore sequence homology alone is not sufficient to determine specific ligand or ligands for each of the receptors. To date two of the group A Tlp receptors, Tlp1 and Tlp7, have been characterised [33]. CcaA was identified as the only receptor conserved in all sequenced strains of *C. jejuni* [34] and determined to be the receptor for aspartate [35] whereas Tlp7 binds to formic acid [36].

In this study we describe the characterisation of the periplasmic sensory domain of Cj1564 (Tlp3) chemoreceptor of *C. jejuni* strain NCTC 11168-O and characterise the ligand binding specificities of this chemoreceptor protein. We demonstrate the multifunctional role of Cj1564 in recognition of chemoattractants as well as chemorepellents, its association with the scaffolding proteins CheV and CheW and demonstrate the role of Cj1564 in cell to cell adhesion (autoagglutination) and biofilm formation, cell shape and host colonisation.

## Results

### Identification of ligand binding specificity of Tlp3

To gain a greater understanding of the *C. jejuni* chemotactic pathway, characterisation of ligand binding potential for Cj1564 (Tlp3) was performed using recombinant Tlp3 periplasmic sensory domain peptide with all 20 amino acids and salts of organic acid arrays as well as 96-well binding assays (Table 1). The array assay presents the compounds covalently bound to an epoxide group resulting in a high probability of a single molecular orientation. The plate assay relies on non-covalent charge and hydrophobic/hydrophilic interactions for presentation of the molecules offering a different presentation to the array. A total of 12 interacting ligands were identified through the plate and array assays. The binding of the Tlp3 sensory domain to amino acids and salts was then confirmed by STD-NMR and SPR (Biacore, Table 1). SPR analysis found the highest affinity interactions of Tlp3 with lysine and glucosamine (K_D_<10 µM; Table 1), additionally, biologically significant interactions (K_D_<50 µM; Table 1, Figure S1) were observed for isoleucine, succinic acid, arginine, purine, malic acid and thiamine.

**Table 1:** Summary of ligand binding and chemotactic response.

Ligand	Array binding	Plate assay	Binding Affinity (µM)	Chemotactic response
Isoleucine	Y	Y	17.75	+
Lysine	N	Y	2.80	−
Glucosamine	ND	Y	7.77	−
Succinic Acid	ND	Y	33.23	−
Arginine	N	Y	38.55	−
Purine	ND	Y	38.28	+
Malic Acid	ND	Y	18.37	+
Fumaric Acid	ND	Y	>10,000	+
Thiamine	ND	Y	38.55	−
Aspartate	N	Y	150.4	+
α-ketoglutarate	Y	Y	295.6	+/−

+, positive response (chemoattractant); −, negative response (chemorepellent); Y, positive binding significantly above background; N, binding not significantly above background; ND, not determined. Other compounds tested in both plate and array assays but showing no binding were: alanine, asparagine, histidine, leucine, methionine, cysteine, phenylalanine, glutamic acid, threonine, glutamine, tryptophan, glycine, valine, proline, serine, tyrosine.

### Mutation of *tlp3* alters motility, autoagglutination and biofilm formation of *C. jejuni*

**Figure 1:**
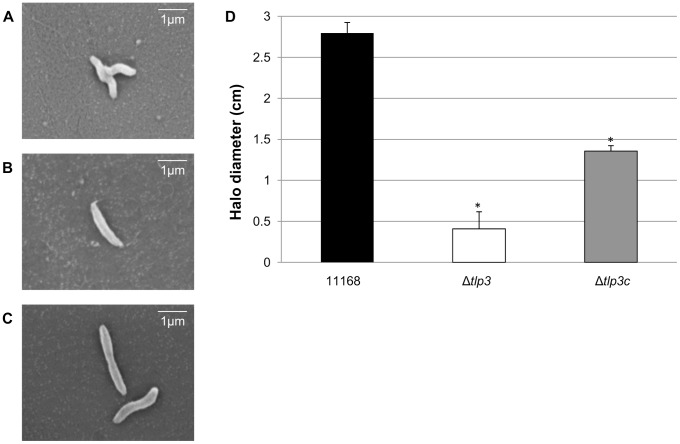
A *tlp3* mutant has altered morphology and defect in motility. Images of; A, helical strain of 11168 original clinical isolate; B, straight Δ*tlp3* strain; C, the complemented strain Δ*tlp3*c with restored helical morphology in ∼50% of the population. Scale bar: 1 µm; D, Δ*tlp3* exhibits a significant motility defect as assayed using halo diameter on soft agar plates. Standard errors of the mean were calculated from three independent experiments. The asterisk (*) indicates a statistically significant difference compared to wild type 11168-O using the unpaired Student's t-test, p<0.01.

*Cj1564* (Tlp3) has previously been identified as a group A chemoreceptor for *C. jejuni* with homology in the signalling domain to other bacterial chemoreceptors such as that of *E. coli* and *H. pylori* [37]. The periplasmic domain of *C. jejuni* group A chemoreceptors are predicted to be involved in sensing and binding of extracellular ligands [37]. In order to identify biologically significant ligand binding specificity and function of Tlp3 in the chemotactic pathway, an insertionally inactivated isogenic *tlp3* mutant and its complement were created. The mutant was constructed by deleting 52 central bp of the periplasmic domain of *tlp3* and inserting a non-polar kanamycin resistance cassette which has the transcription terminator removed to minimise effects on genes downstream that may be transcribed in the same orientation as *tlp3* (described in materials and methods: mutagenesis and complementation of *tlp3*). Microscopic analysis of Δ*tlp3* confirmed the presence of flagella and altered (loss of spiral shape) cellular morphology of all observed 11168-O Tlp3 mutant bacterial cells, with spiral morphology restored after complementation in approximately 50% of the complemented cells (Figure 1A, B & C). Comparison of *C. jejuni* 11168–O and Δ*tlp3* motility shows that there was a 5-fold decrease in non-directed swimming motility of the mutant with motility partially restored to wild type levels in the complemented strain, Δ*tlp3*c (Figure 1D). Live imaging of fluorescently labelled *C. jejuni* isogenic strains allowed capture of bacterial cell motility and demonstrated a defect in motility illustrated by the inability of Δ*tlp3* cells to effectively ‘swim’ in a directional movement in absence of specific stimuli. Instead the cells were observed to ‘twitch’ and continuously ‘tumble’ without movement in any particular direction (Movie S1), thus demonstrating an altered “random walk” motility phenotype of the Δ*tlp3* mutant. The expression of the *tlp3* gene in the isogenic mutant strain was reduced, as compared with the wild type and complemented strains, which is likely to be due to the interruption of the gene with the strongly promoted Km^R^ cassette. Expression of *tlp3* was analysed using quantitative real time PCR in 11168-O, Δ*tlp3* and Δ*tlp3*c using primers that amplify the entire periplasmic domain gene region of *tlp3*. There was a 6.5±0.78 fold reduction observed in the expression of *tlp3* in the Δ*tlp3* mutant strain compared to the wild type strain, conversely an 8.75±0.98 fold increase was observed when screening for *tlp3* expression in the complemented mutant compared to wild type (data not shown). This indicates that the inclusion of the resistance cassette into the *tlp3* gene has negatively affected the expression of *tlp3* from its own promoter. This indicated that the Kanamycin resistance cassette encodes the dominant promoter signal that inhibits production of RNA molecules containing the 5′ end of the *tlp3* gene region, upstream from the kanamycin resistance cassette. While the insertion of complementing *tlp3* and chloramphenicol resistance cassette into the pseudogene (*cj0046*) results in over expression of *tlp3* compared to the wild type strain. Further expression analysis was performed on the genes immediately upstream (*cj1563c*) and downstream (*pflA*) of the mutated *tlp3* gene to confirm no polar effects were introduced as a result of mutagenesis. Expression of *pgp1* and *flaA* were assessed to establish that the observed defect in motility is not due to changes in expression of genes involved in flagella development or function. No significant difference in expression (p>0.1) was observed for *cj1563c*, *pflA*, *pgp1* and *flaA* (data not shown).

**Figure 2:**
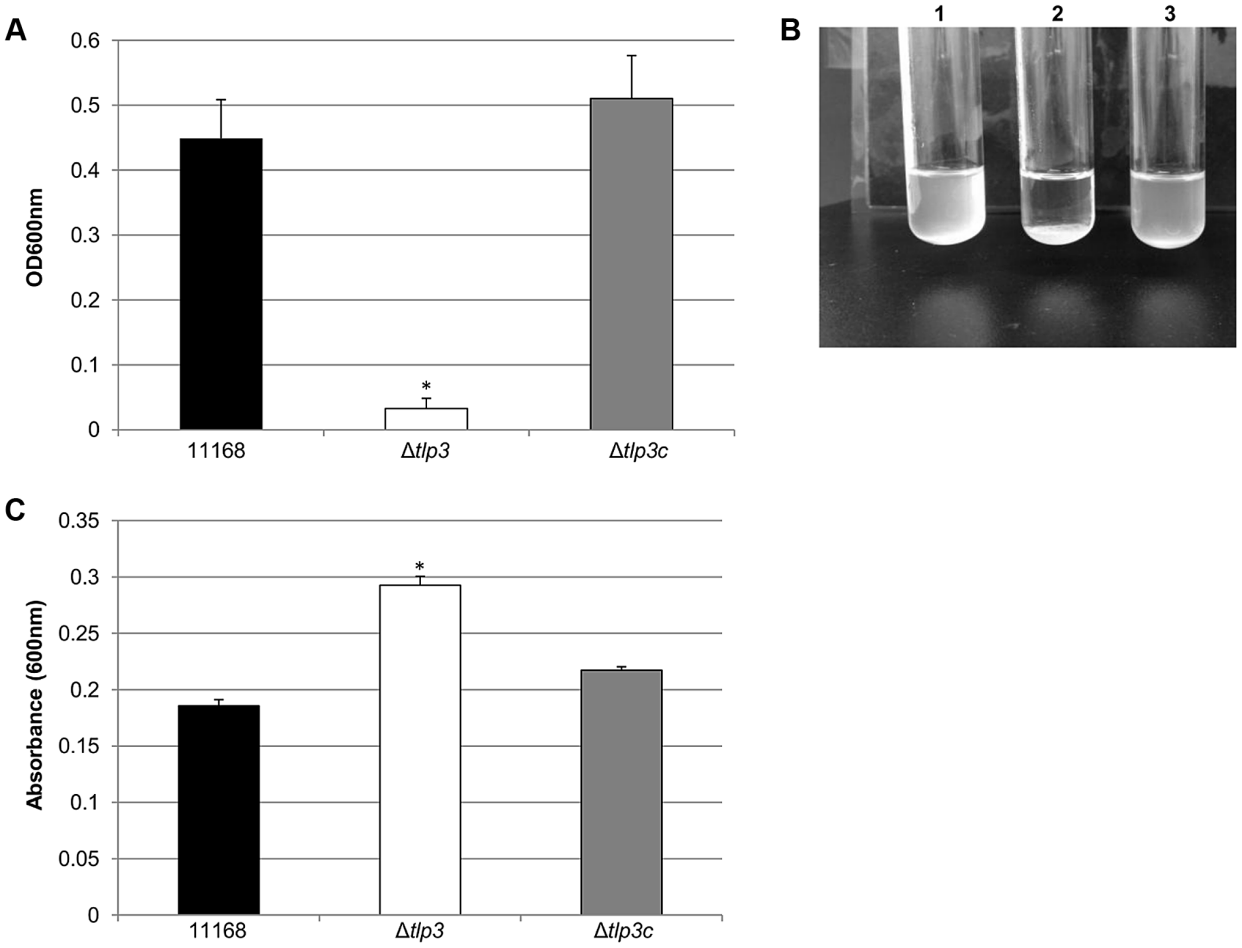
Δ*tlp3* displays altered autoagglutination and biofilm formation. A, autoagglutination of 11168 original clinical isolate, Δ*tlp3* and Δ*tlp3*c strains measured at OD_600 nm_; B, Image of autoagglutination in static suspension after 24 h in strains (1) 11168-O, (2) Δ*tlp3* and (3) Δ*tlp3*c; C, biofilm formation was assessed by crystal violet staining and quantification of dissolved crystal violet at OD_600 nm_. Standard errors of the mean were calculated from triplicate cultures and are representative of three independent experiments. The asterisk (*) indicates a statistically significant difference using the unpaired Student's t-test, p<0.05.

Biofilm formation and agglutination play an important role in the survival of bacterial cells. Consequently, we compared these characteristics between the wild type (11168-O), mutant (Δ*tlp3*) and complemented (Δ*tlp3*c) strains. Autoagglutination assays revealed that the Δ*tlp3* autoagglutinated while 11168-O and Δ*tlp3*c had little to no autoagglutination (Figure 2A & B). It is also interesting to note that autoagglutination of the 11168-O *Δtlp3* mutant was similar to that of *C. jejuni* 81–176 (Figure S2), which carries a natural mutation in its *tlp3* gene, revealed by the published genome sequence (Sanger, 2006). Furthermore, autoagglutination was also shown to be independent of growth temperature at 25°C, 37°C and 42°C for all strains tested (data not shown). Additionally it appears that Δ*tlp3* autoagglutinates at a rate that is faster than normal gravitational pull. Biofilm formation, as assessed by crystal violet assay, indicated an approximate 1.5-fold increase in biofilm formation of Δ*tlp3* mutant compared to wild type, with the original levels in biofilm formation restored in Δ*tlp3*c (Figure 2C).

### Chemotactic behaviour and influence of Tlp3

**Figure 3:**
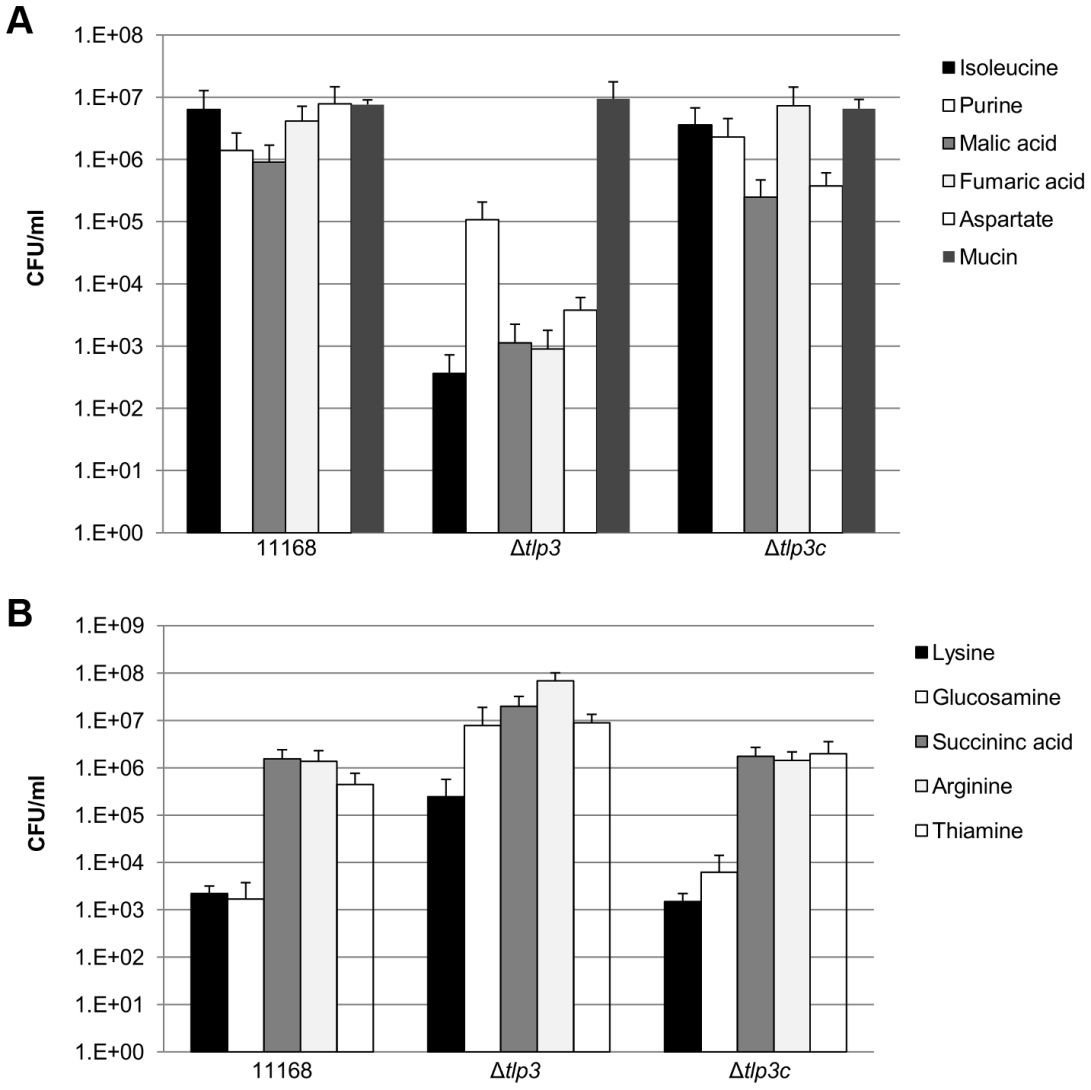
Chemotaxis assay. Viable counts of *C. jejuni* associated with plugs containing; A, ligands considered attractants; isoleucine, purine, malic acid, fumaric acid, aspartate and mucin; B, ligands considered repellents; lysine, glucosamine, succinic acid, arginine and thiamine. A *C. jejuni* non-motile, non-chemotactic mutant (81116Δ*flaA*/*flaB*) control was used along with agar plugs containing no amino acid (negative control) and mucin (positive control). Standard errors are shown as bars above the mean of three replicates.

In order to determine the biological relevance of Tlp3 in chemotactic motility, nutrient depleted chemotaxis assays were performed as previously described [35]. Chemotaxis towards a range of amino acids, glycans and other small molecules was investigated in 11168-O, Δ*tlp3* and the complemented mutant, Δ*tlp3*c. A positive chemotaxis response was identified for 5 amino acids, suggesting these ligands are attractants (Figure 3A; Table 1). A 4-log reduction in bacterial numbers was observed for the Δ*tlp3* (3.6 × 10^2^ cfu/ml) compared to the wild type (6 × 10^6^ cfu/ml) in migration towards isoleucine, additionally this was also observed for fumaric acid (9 × 10^2^ and 4.14 × 10^6^ cfu/ml respectively). For migration towards purine, a 1-log reduction for the Δ*tlp3* (1.08 × 10^5^ cfu/ml) compared to the wild type (1.4 × 10^6^ cfu/ml) was observed, with a 2-log reduction in migration towards malic acid (1.13 × 10^3^ and 9 × 10^5^ cfu/ml respectively) and a 3-log reduction in migration towards aspartate (3.7 × 10^3^ and 7.8 × 10^6^ cfu/ml respectively). The migration of wild type *C. jejuni* 11168-O towards aspartate was comparable to that previously published for nutrient depleted assay by Hartley-Tassell *et al.*, 2010 [38]. Tlp3 was also found to mediate a repellent response to 5 amino acids (Figure 3B; Table 1). Lysine mediated repellence was reduced for the Δ*tlp3* isogenic strain as a 2-log increase in viable bacterial numbers was detected around the lysine plug (2.4 × 10^5^ cfu/ml) when compared to the wild type (2.2 × 10^3^ cfu/ml). For migration of Δ*tlp3* towards glucosamine, a 3-log increase (7.8 × 10^6^ cfu/ml) was detected, compared to that of the wild type (1.68 × 10^3^ cfu/ml). In addition, for succinic acid, arginine and thiamine, a 1-log increase of bacterial numbers was observed in the Δ*tlp3* (2 × 10^7^, 6.8 × 10^7^ and 9 × 10^6^ cfu/ml, respectively) compared to the wild type strain (1.5 × 10^6^, 1.3 × 10^6^ and 4 × 10^5^ cfu/ml, respectively). Chemotaxis assay for alpha-ketoglutarate was not definitive (data not shown), possibly due to the low affinity of this receptor for alpha-ketoglutarate.

### Identification of chemoattractant and chemorepellent binding of Tlp3 by STD-NMR analysis

**Figure 4:**
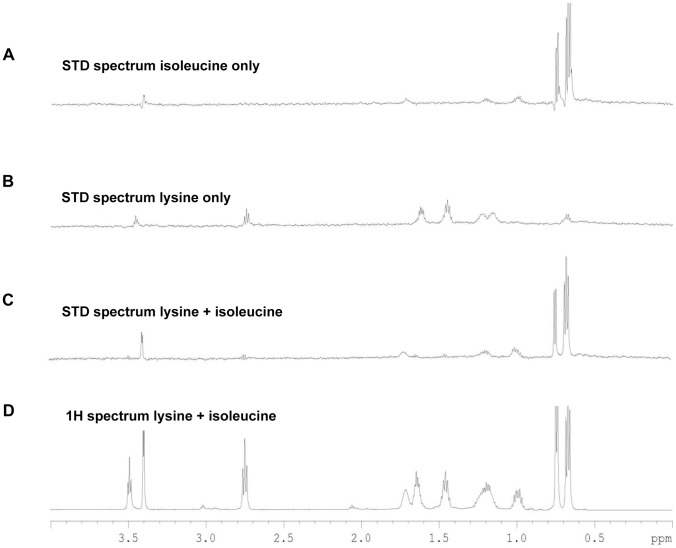
STD-NMR analysis spectra for Tlp3. A, STD spectrum of isoleucine/Tlp3; B, STD spectrum of lysine/Tlp3; C, STD spectrum of lysine and isoleucine (competitive)/Tlp3; D, ^1^H NMR spectrum of lysine and isoleucine in the presence of Tlp3. All STD spectra are scaled × 64 compared to the ^1^H spectrum. All spectra were recorded at 283 K and 600.13 MHz.

^1^H STD-NMR analysis was used to investigate the epitope binding preferences of the chemoattractants (isoleucine, purine, malic acid) and chemorepellents (lysine, arginine, glucosamine) to the recombinant Cj1564 (Tlp3) periplasmic sensory domain peptide. Binding was observed with chemoattractants isoleucine and purine along with the chemorepellents lysine and arginine in accordance with the SPR analysis showing varying binding affinities. For the chemoattractant isoleucine (K_D_∼17 µM), the binding epitope appeared to be the methyl group, as strong methyl resonances were seen in the STD spectrum and only very weak signals for other side chain resonances (Figure 4A). For purine (K_D_∼38 µM), the H6 and to a lesser extent the H2 protons (∼5-fold) of the pyrimidine ring showed an STD effect compared to a negligible STD effect for the H8 proton of the imidazole ring. For the chemorepellent lysine (K_D_∼2.8 µM) an STD effect was clearly seen for all proton resonances along the side chain (Figure 4B). A weaker STD effect (∼4 fold) was seen for all proton resonances along the arginine (K_D_∼38 µM) side chain. No signals were detected for malic acid or glucosamine using STD-NMR. SPR analysis demonstrated that glucosamine and malic acid have slow disassociation rates explaining the absence of STD signals due to these amino acids as saturation transfer is a result of the spin diffusion process which requires fast exchange for magnetisation to be spread from the protein to the ligand in order to observe an STD effect.

Competition STD experiments were also performed to probe the binding preferences of Tlp3 and to unravel the nature of the binding interaction. Competition experiments testing the effect on binding to Tlp3 of attractant/attractant (isoleucine/purine), repellent/repellent (lysine/arginine) and attractant/repellent (isoleucine/arginine; isoleucine/lysine; purine/lysine; purine/arginine) were performed. For attractant/attractant (isoleucine K_D_∼17 µM/purine K_D_∼38 µM) there seemed to be a minor change in the STD effect seen for the ligands independently. For the repellent/repellent (lysine K_D_∼2.8 µM/arginine K_D_∼38 µM) however, there appeared to be a significant reduction in the STD effect of lysine in the presence of arginine whose weak STD effect appeared unchanged, even though the binding affinity of lysine is stronger (∼14 fold) than that of arginine. These results demonstrate that Cj1564 (Tlp3) is able to bind to both chemoattractants and chemorepellents. No binding preference to Cj1564 (Tlp3) was observed in the presence of the chemoattractants isoleucine/purine, compared with the ligands alone, however for the chemorepellent competition experiment even though lysine has a greater binding affinity (K_D_∼2.8 µM) compared with isoleucine (K_D_∼17 µM) a significant reduction in the STD effect of lysine was observed in the presence of arginine suggesting preferential binding of arginine over lysine.

In the case of the attractant/repellent spectra: Isoleucine/arginine - isoleucine (chemoattractant, K_D_∼17 µM) binds preferentially over arginine (chemorepellent, K_D_∼38 µM). For isoleucine/lysine - isoleucine (chemoattractant) binds preferentially over lysine (chemorepellent) with a significant reduction in the STD effect of lysine observed in the presence of isoleucine compared to the STD spectra of the ligands individually, even though the binding affinity of lysine (K_D_∼2.8 µM) is stronger than that of isoleucine (K_D_∼17 µM) (Figure 4C & D). Purine/lysine - purine (chemoattractant) binds preferentially over lysine (chemorepellent) with a significant reduction in the STD effect of lysine in the presence of purine observed compared to the STD spectra of the ligands individually, even though the binding affinity of lysine (K_D_∼2.8 µM) is stronger than that of purine (K_D_∼38 µM). Purine/arginine - purine (chemoattractant) binds preferentially over arginine (chemorepellent), a negligible STD effect of arginine in the presence of purine was observed with these ligands determined to have comparable binding affinities (K_D_∼38 µM). These results demonstrate that there is preferential binding of chemoattractants over the chemorepellents regardless of the individual binding affinity of the ligands to Cj1564 (Tlp3).

### Involvement of *tlp3* in host bacterial interactions

#### *In vitro* invasion in the epithelial cell line Caco-2 is significantly reduced in Δ*tlp3*

**Figure 5:**
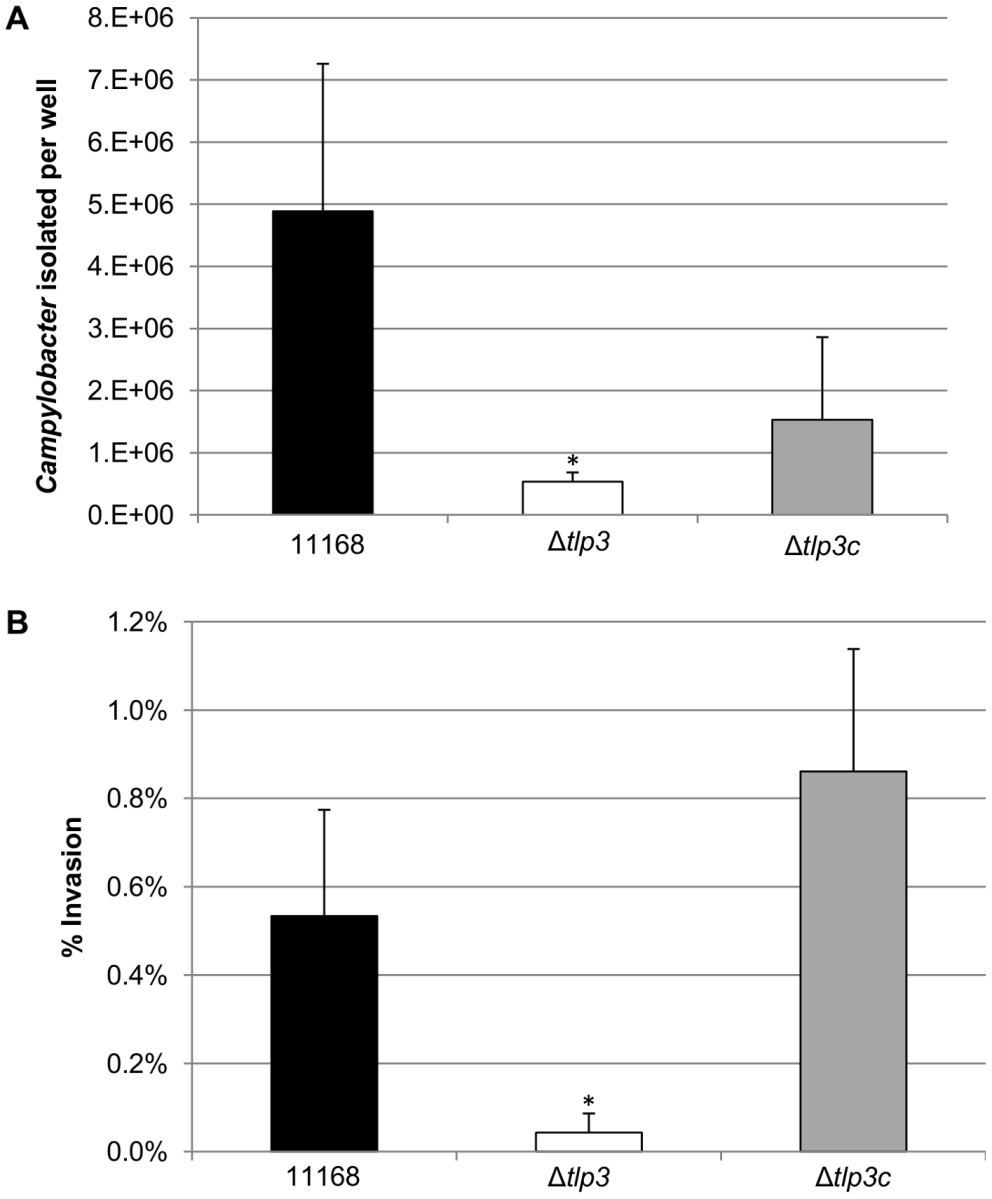
The role of Δ*tlp3* on host phenotype. A, *In vitro* adherence analysis represented as the mean of total adherent-plus-invaded bacteria; B, *In vitro* invasion analysis represented as percentage of invading bacteria with respect to total adherent bacteria. Standard errors of the mean were calculated from triplicate cultures and are representative of three independent experiments. The asterisk (*) indicates a statistically significant difference using the unpaired Student's t-test, p<0.05.

The intestinal epithelial cell line Caco-2 was used in the gentamicin protection assay to assess the role of Tlp3 in attachment and subsequent invasion *in vitro* using an isogenic Δ*tlp3* and Δ*tlp3*c strain. The isogenic Δ*tlp3* mutant displayed a 6-fold reduced level of adherence and 13-fold reduction in invasion of the human intestinal Caco-2 cell surface, compared to the wild type strain or complemented mutant strain Δ*tlp3c* (Figure 5A and 5B). This finding further suggests that a mutation in *tlp3* affects the ability of the bacteria to actively adhere and invade cultured epithelial cells.

#### Δ*tlp3* does not affect chick colonisation

The effect of *tlp3* deletion from the *C. jejuni* 11168-O chemoreceptor repertoire *in vivo* was explored using a *C. jejuni* chick colonisation model [39]. One-day old chicks were infected orally with a dose of 10^8^ *C. jejuni* wild type 11168-O, Δ*tlp3* or Δ*tlp3*c, and caecal contents were assessed for *C. jejuni* after 5 days post infection, once complete colonisation had been reached [39]. The Δ*tlp3* isogenic mutant strain exhibited no significant difference in colonisation ability compared to wild type (Figure S3). This interesting result, together with the increased Δ*tlp3* autoagglutination data, led us to hypothesise that the Δ*tlp3* mutant may aggregate on the surface of the caeca. To confirm this, we used scanning electron microscopy to visualise Δ*tlp3* isogenic mutant strain cells *in vivo* on the chicken caeca. Figure S4A shows *C. jejuni* 11168-O wild type cells loosely scattered on the surface of the caecal tissue while Figure S4B shows a tightly compacted aggregate of Δ*tlp3* isogenic mutant cells. As there was no difference between colonisation ability of the wild type bacteria and Δ*tlp3*, we can speculate that *in vivo* in an avian host, the Δ*tlp3* may be able to detect a chemotactic signal to follow in the lumen or caeca, allowing subsequent aggregation of the bacteria on the caecal surface coincidently resulting in similar colonisation levels.

### The signalling domain of Tlp3 interacts with other proteins and protein domains of the *C. jejuni* chemotaxis signalling pathway

To further investigate the role of Tlp3 in the chemotaxis signal transduction pathway in *C. jejuni*, the predicted cytoplasmic signalling domain of Tlp3 was analysed for protein-protein interactions with *C. jejuni* NCTC11168-O chemotaxis proteins using the yeast two-hybrid and three-hybrid systems as previously described [35]. The yeast two-hybrid system was used because of a lack of similar genetic manipulation systems available for *C. jejuni*. We have previously demonstrated biological validity of protein interactions identified by the yeast two-hybrid system via pull-down assays for the components of the *C. jejuni* aspartate receptor, CcaA [35] signal transduction. The yeast system allows detection of interacting proteins *in vivo* by utilising the two separable domains of the GAL-4 transcription factor, the DNA-binding domain (DNA-BD) and the transcription activation domain (AD). This system relies on the reconstitution of the GAL-4 transcription factor when two proteins of interest, the ‘bait’ protein fused to the DNA-BD and the ‘target’ protein fused to the AD, interact, thus allowing activation of reporter gene expression [40], Residues 517–662 of Tlp3, encompassing the region homologous to the highly conserved MCP signalling domain, were selected for analysis. The signalling domain is an independent structural motif, which interacts with the dimeric CheA and the scaffolding protein CheW to form MCP-CheW-CheA ternary signalling complexes that regulate CheA histidine kinase activity [41]–[43]. It is necessary to note that this region of Tlp3 is identical to residues 513–659 of Tlp2 (Cj0144) and 520–665 of Tlp4 (Cj0262) [37] and thus is referred to as Tlp^sig^_2.3.4_ in this study (Figure S5). The Tlp^sig^_2.3.4_ was found to interact with a number of the *C. jejuni* NCTC11168-O chemotaxis proteins and protein domains, as shown in Table 2. A medium strength interaction was detected between Tlp^sig^_2.3.4_ and CheV (AD-Tlp^sig^_2.3.4_ with BD-CheV). An identical interaction was observed in the reciprocal comparison of fusion proteins (AD-CheV and BD-Tlp^sig^_2.3.4_). The Tlp^sig^_2.3.4_ was found to bind only to the CheW-like domain of CheV (CheV^dW^). A medium strength interaction was detected between these domains in reciprocal comparisons (AD-Tlp^sig^_2.3.4_ with BD-CheV^dW^ and AD-CheV^dW^ and BD-Tlp^sig^_2.3.4_) while no interaction was detected with the response regulator domain of CheV (CheV^dRR^ and Tlp^sig^_2.3.4_). Two-hybrid analysis also revealed that the Tlp^sig^_2.3.4_ signalling domain was capable of dimerisation (AD-Tlp^sig^_2.3.4_ and BD-Tlp^sig^_2.3.4_). An interaction was also observed between Tlp^sig^_2.3.4_ and CheW. This interaction was of a medium strength (AD-CheW and BD-Tlp^sig^_2.3.4_); however this interaction was not detected with the reciprocal combination of fusion proteins.

**Table 2:** Analysis of protein interactions with Tlp^sig^_2.3.4_ using the yeast two-hybrid system.

AD- Tlp^sig^_2.3.4_ with:		BD- Tlp^sig^_2.3.4_ with:	
BD- Tlp^sig^_2.3.4_	++	AD- Tlp^sig^_2.3.4_	++
BD-CheA	0	AD-CheA	0
BD-CheA^dHK^	0	AD-CheA^dHK^	0
BD-CheA^dRR^	0	AD-CheA^dRR^	0
BD-CheW	0	AD-CheW	++
BD-CheV	++	AD-CheV	++
BD-CheV^dW^	++	AD-CheV^dW^	++
BD-CheV^dRR^	0	AD-CheV^dRR^	0
BD-CheY	0	AD-CheY	0
BD-CheB	0	AD-CheB	0

Testing for interactions of Tlp^sig^_2.3.4_ fused to the GAL-4 activation domain (AD- Tlp^sig^_2.3.4_) and GAL-4 binding domain (BD- Tlp^sig^_2.3.4_) with all chemotaxis proteins and individual domains fused to the GAL-4 DNA binding domain and GAL-4 activation domain respectively. Co-transformation of AD-CheW and BD-CheA was used as a positive control when testing for interactions, with a result of +++.

+++: cream, dense growth of >75% of co-transformants observed on high stringency media and intermediate stringency media.

++: cream − pink moderate growth for ∼75% or more of co-transformants observed on high stringency media and intermediate stringency media.

+: cream − pink light growth for ∼50% or more of co-transformants observed on high stringency media and/or intermediate stringency media.

0: no growth of co-transformants observed.

As it has previously been shown that CcaA (Tlp1) of *C. jejuni* preferentially binds to CheV over CheW [35], we utilised the yeast three-hybrid system to determine if the signalling domain of Tlp3 (which is identical to the signalling domains of Tlp2 and Tlp4) displayed similar binding preferences for the scaffolding proteins. In the three-hybrid system, a third protein can be conditionally expressed and its role in the interaction between the AD and DNA-BD fusion proteins can be examined [44]. We specifically looked at the effect of presence of both CheW and CheV proteins on their interactions with Tlp^sig^_2.3.4_. It was found that the co-expression of native CheW from pBrTlp234IWII (Table 3) had no effect on the medium strength interaction observed between Tlp^sig^_2.3.4_ and CheV (BD-Tlp^sig^_2.3.4_ and AD-CheV). Analysis of interactions between the reciprocal combinations of fusion proteins using pBrVIWII (Table 3) showed that the co-expression of native CheW slightly enhanced the medium strength interaction of CheV with Tlp^sig^_2.3.4_ (BD-CheV and AD-Tlp^sig^_2.3.4_). When protein interactions of Tlp^sig^_2.3.4_ with CheW were investigated with native CheV co-expressed (pBrTlp234IVII), it was found that the presence of CheV strengthened the weak interaction observed between the Tlp^sig^_2.3.4_ signalling domain and CheW (BD-Tlp^sig^_2.3.4_ and AD-CheW). This was confirmed in the analysis of the interactions between the reciprocal combination of fusion proteins (AD-Tlp^sig^_2.3.4_ and BD-CheW) using pBrWIVII.

**Table 3:** Analysis of protein interactions with Tlp^sig^_2.3.4_ using the yeast three-hybrid system.

BD- Tlp^sig^_2.3.4_ with:	Expression of third protein (CheW) repressed	Expression of third protein (CheW) induced
AD-CheW	0	0
AD-CheV	++	++
AD- Tlp^sig^_2.3.4_	0	0

	Expression of third protein (CheV) repressed	Expression of third protein (CheV) induced
AD-CheW	+	++
AD-CheV	++	++
AD- Tlp^sig^_2.3.4_	++	++

BD-CheV with:	Expression of third protein (CheW) repressed	Expression of third protein (CheW) induced
AD- Tlp^sig^_2.3.4_	+/++	++

BD-CheW with:	Expression of third protein (CheV) repressed	Expression of third protein (CheV) induced
AD- Tlp^sig^_2.3.4_	0	+

+++ (strong): dense, cream growth for all co-transformants on His deficient media.

++ (medium): moderate, cream-pink growth for ∼75% of co-transformants on His deficient media.

+ (weak): minimal, pink-red growth for ∼50% of co-transformants on His deficient media.

0: no growth of co-transformants observed on His deficient media.

## Discussion

In this study we have shown the role of Tlp3 in *C. jejuni* 11168-O chemotaxis as a multiple ligand-binding protein, capable of detecting numerous chemoattractants and chemorepellents using a range of confirmatory methodologies such as small molecule arrays, SPR and STD-NMR. The substrates identified as having a strong binding affinity include lysine and glucosamine with biologically significant interactions observed for isoleucine, aspartate, succinic acid, arginine, purine, malic acid, and thiamine. Therefore we propose to name this receptor CcmL; *Campylobacter* chemoreceptor for multiple ligands.

Similar observations have previously been made for *H. pylori* isolates, where positive chemotaxis was identified for the sugars and amino acids phenylalanine, aspartic acid, glutamic acid, isoleucine and a negative chemotaxis for leucine and tyrosine [45]. Isoleucine has previously been demonstrated to be a chemoattractant for other organisms including *B. subtilis* [46], *H. salinarum* [47], *P. aeruginosa* [48] and *H. pylori* [45]. In *B. subtilis*, the McpC chemoreceptor binds isoleucine weakly, yet with sufficient affinity to suggest direct binding. It is interesting to note that in our study, glucosamine has been identified as a repellent, while in other bacteria such as *E. coli* [49] and *B. burgdorferi* it has been determined to be an attractant, and has been classified as a non-essential nutrient [48], [50]. Lysine has been demonstrated as an attractant for *P. aeruginosa* [48] which is opposite to our findings which show lysine as a chemorepellent for *C. jejuni*. Furthermore, in *B. subtilis*, the McpC chemoreceptor did not show binding to lysine, however, it was suggested that McpC binds lysine by an indirect method most likely involving ancillary proteins, further suggesting McpC may be a universal chemoreceptor able to respond to numerous amino acids [51]. It had been shown in *E. coli* that even though chemoreceptors are sensitive to a particular ligand, they can also detect a large number of structurally related amino acids and their analogues [52].

*C. jejuni* is highly adapted to the environment of the avian gut and as a result, uses efficient chemotactic motility to colonise the mucous-filled crypts of the lower gastrointestinal tract [53]. It is reasonable to hypothesise that Tlp3 may be involved in interacting with ligands to sense the external environment in order to navigate inside the host and may be involved directly or indirectly in *C. jejuni* metabolic and catabolic pathways. It is interesting to note that aspartate, glutamate, proline and serine are the most abundant amino acids found in chicken excreta [54], and serine catabolism has been reported to be essential for colonisation of the avian gut by *C. jejuni* [55]. However, *C. jejuni* lacks the key glycolytic enzyme 6-phosphofructokinase as well as alternative pathways for sugar catabolism [56] so it utilises amino acids and Krebs cycle intermediates for energy production and encodes all of the enzymes required for a complete oxidative TCA cycle [37], [57], [58]. *C. jejuni* needs to be able to sense and move towards amino acids and small organic acids such as aspartate, asparagine and serine in order to catabolise these compounds to use as the sole source of reduced carbon and energy due to its inability to utilise glucose [59]. Additionally, a recent study has indicated that energy taxis may also be one of the driving forces behind movement to optimal conditions for energy generation and subsequent colonisation [60], [61].

We have shown that a mutation in the *C. jejuni ccmL* chemoreceptor gene lead to alteration of phenotypic characteristics of the bacteria, such as cellular morphology, autoagglutination behaviour and biofilm formation, highlighting its role in the *C. jejuni* life cycle. Furthermore, the signalling domain of this chemoreceptor also interacts and binds with both CheV and CheW scaffolding proteins.

Additionally, we used STD-NMR analysis to confirm the binding of amino acid and salts of organic acid arrays, plate binding assays and SPR analysis. STD-NMR indicated that the Tlp3 receptor binds chemoattractants preferentially over chemorepellents irrespective of binding affinity, however, is still able to recognise both attractants and repellents in isolation. This may indicate that upon binding an attractant, the repellent binding site of CcmL through allostery prevents the binding of a repellent ligand to the protein. Furthermore, it appears that the binding site may be able to accommodate more than one chemoattractant at a time as competition tests between two chemoattractants results in positive binding for both attractants present; indicating that one chemoattractant is not preferred over another regardless of the measured affinity. These results indicate a much more complex interaction between chemotaxis receptor proteins and ligands than previously thought, with a complex milieu of attractants acting in concert rather than the receptor binding preferentially to a specific ligand based on the hierarchy of affinities. Furthermore, the Tlp3 predicted structure indicates the presence of a single Cache_1 (PFO2743) (calcium channels and chemotaxis receptors) domain, representing a single ligand binding pocket that accommodates multiple ligands with varying affinity. This is in agreement with our STD-NMR data that shows interchange of the ligands binding to purified periplasmic domain of CcmL in competitive assays. CcmL (Cj1564) shares complete homology in the cytoplasmic domain with Tlp2 (Cj0144) and Tlp4 (Cj0262), but has less than 50% homology with the cytoplasmic domains of Tlp1 (Cj1506), Tlp7 (Cj0951/52) and Tlp10 (Cj0019). Tlp3 has the greatest homology to Tlp2 with 72% identity across the entire protein and 38% identify/59% similarity across the periplasmic domain. The greatest stretch of similarity between Tlp3 and Tlp2 in the periplasmic binding region is within the cache domain that is present in both proteins. Homology between the periplasmic domain of Tlp3 and the periplasmic domains of Tlp1 and 4 is below 33%. No homology was detected between Tlp3 periplasmic domain and the periplasmic domains of Tlp7 and 10.

To further characterise interactions of Tlp3 with components of the *C. jejuni* chemotaxis signalling pathway, a well-established yeast-two-hybrid and three-hybrid protein-protein interaction system was used. The two- and three-hybrid system data suggests that both the scaffolding proteins CheW and CheV are capable of binding to the Tlps2, 3 and 4 signalling domains with no obvious preference for either protein observed. CheW and CheV may form mixed multi-protein complexes with these receptors, as the expression of CheV was found to strengthen the interaction between the signalling domain of Tlp^sig^_2.3.4_ and CheW. This appears to vary from the observations for the CcaA (Tlp1) signalling domain, which has previously been shown to preferentially bind CheV [35]. While the signalling domains of Tlps 2, 3 and 4 and of Tlp1 are very similar, some amino acid differences do exist which may account for the differences in the binding preferences of these Tlps for the CheW and CheV scaffolding proteins. These amino acid differences are within the region homologous to residues 350–471 of the *E. coli* serine chemoreceptor, Tsr, a fragment which has been shown to be capable of mediating CW-biased signals and is therefore predicted to contain residues involved in the binding of CheW and CheA in this species [62] (Figure S5). This data lead us to speculate that in *C. jejuni*, the group A Tlp2, 3 and 4 signal via alternative or mixed scaffolding proteins and that different binding affinities of the chemoreceptors with CheV and CheW may control the composition of receptor clusters.

The mutation in the *tlp3* (*cj1564*) gene resulted in an altered cellular morphology of the bacterial cell and inability of Δ*tlp3* cells to ‘run’. Increased formation of biofilm and the high rate of autoagglutination may indicate a possible increased response to stress by the *tlp3* mutant. A previous study by Vegge *et al.* (2009) reported that mutation of the *tlp3* gene along with other genes does not affect the motility of *C. jejuni* in a rich medium [63] however; our results indicate mutation of *tlp3* significantly reduces motility when compared to the wild type and complemented strains. This is in agreement with a study carried out by Golden *et al.* (2002) [64] where mutation of *tlp3* reduced motility and complementation restored the wild type phenotype [23]. Furthermore, reduced motility is also likely due to an inability of the bacteria to engage in efficient chemotaxis signalling. Further inspection of the isogenic Δ*tlp3* mutant cells by microscopy indicated that the bacteria retained the ability to twitch and tumble in a stationary position suggesting a bias toward clockwise rotation of the flagella, subsequently leading to a higher frequency of tumbles and inhibited smooth swimming. It appears however, that the bacterial cell was still capable of directional movement when a stimulus was supplied *in vivo*. Additionally, scanning electron microscopy and quantitative PCR analysis confirmed the presence of flagella indicating that the lack of motility was not due to a defect in the flagella development; however, the helical shape of the bacteria was altered. The helical shape of *C. jejuni* has long been associated with pathogenesis but the genetic components involved in modulating *C. jejuni* morphology have only recently been identified, where the protein termed Pgp1 (peptidoglycan peptidase 1) in *C. jejuni* 81–176 was identified to be involved in maintenance of the helical shape [65]. To date no correlation has been found between chemoreceptors and cellular morphology.

In the *in vitro* model of infection, it appears that Tlp3 may be a critical factor for invasion as Δ*tlp3* displayed a markedly reduced level of adherence and invasion compared to the wild type strain. However, *in vivo* assessment of the Δ*tlp3* mutant and wild type strain showed no differences in colonisation ability of the avian caeca based on cell counts, even though ability to adhere and invade caco-2 cells *in vitro* was significantly reduced. This finding agrees with the previously published data on avian colonisation for the Tlp3 mutant of *C. jejuni* 81–176 [66]. However, it is important to consider that subsequent publication of the genome sequence of 81–176 (Sanger, 2006) revealed a natural mutation of the Tlp3 homologue in 81–176 which exists as 2 separate reading frames: CJJ81176_1548 and CJJ81176_1549 encoding the majority of the periplasmic and cytoplasmic domains of Tlp3, respectively. There is a nucleotide deletion in the sequence of CJJ81176_1548 at position 1467624 (A) in the genome subsequently altering the amino acid sequence, consequently changing the reading frame of the transmembrane and cytoplasmic domains (CJJ81176_1549) resulting in incorrect translation and production of a non-functional Tlp3 protein in 81–176. There are an additional number of mutations in both the remainder of the periplasmic and cytoplasmic domains with several intervening stop codons. The absence of a functional Tlp3 in 81–176 has previously been reported [67].

It is interesting to note that *C. jejuni* 81–176 has the same complement of Tlp genes as 11168 and 81116, with the exception of Tlp3, which has a naturally occurring mutation in 81–176. When phenotypic characteristics pertaining to biofilm formation and autoagglutination of 81–176, and 11168-O are compared, a wild type 81–176 shows higher levels of aggregation, biofilm formation (data not shown) and autoagglutination, similar to that observed for Tlp3 mutant of 11168 (Figure S2).

Characterising the function of *C. jejuni* chemosensory proteins, as described in this study, will contribute to understanding chemotaxis signalling pathways which are involved in colonisation and further identify chemoreceptor ligand specificity of individual group A Tlp receptors and their involvement in the chemotaxis pathway and its importance in the survival of this organism. The findings in this study also provide insight into the complexity of chemotaxis receptor protein-ligand interactions with implications not just for *C. jejuni* chemotaxis but for all bacterial chemotaxis.

## Materials and Methods

### Ethics statement

Animal experiments were carried out in strict accordance with the Griffith University Animal Ethics Committee guidelines and assigned approval number BDD/02/11. All procedures involving animals were reviewed and approved by National Health and Medical Research Council Australian code of practice for the care and use of animals for scientific purposes 7th edition 2004.

### Bacterial strains, growth conditions and plasmids

Bacterial strains, yeast strains and plasmids used in this study are described in Table S1. *C. jejuni* strain 11168-O from Skirrow collection was kindly provided by DG Newell, Central Veterinary Laboratories, UK. *C. jejuni* strains were grown at 37°C or 42°C on Columbia agar supplemented with 5% defibrinated horse blood (HBA) with vancomycin (10 µg/ml), trimethoprim (2.5 µg/ml) and polymyxin B (2.5 IU/ml) under microaerobic conditions (5% O_2_, 10% CO_2_, 85% N_2_) for 18–24 h. Strains with plasmids for mutation or complementation studies were grown with 50 µg/ml kanamycin (Km) or 30 µg/ml chloramphenicol (Cm). Host *E. coli* BL21 DE3 (Novagen, USA) and *E. coli* DH5α (Novagen, USA) strains were grown in Luria-Bertani (LB) medium (Oxoid) supplemented with ampicillin (100 µg/ml), kanamycin (50 µg/ml) and chloramphenicol (30 µg/ml) when required. Host yeast strains were grown and prepared according to manufacturers' instructions (Clontech). Labelling of bacterial cells with CFDA-SE was performed as previously described [35], [68].

### Expression and purification of the sensory domain of *tlp3*

The DNA fragment encoding the periplasmic sensory domain (amino acids 43–290) of Tlp3 was amplified using primers incorporating start and stop codons (Table S2) and ligated into pGEM-TEasy to form pGU0815 (Table S1). The insert in pGU0815 was restricted at primer-specific *Nde*I and *Xho*I sites and subcloned into pET-19b to form pGU0816 (Table S1). For recombinant protein expression, competent *E. coli* BL21(DE3) cells were transformed with pGU0816. An overnight culture of BL21(DE3)/pGU0816 was used to inoculate LB containing ampicillin (100 µg/ml) that was incubated at 37°C with aeration. Protein expression was induced using 1 mM IPTG when OD_600 nm_ reached 0.4–0.6. Expression of the Tlp3^peri^ -His fusion protein was verified by SDS-PAGE and Western Blot analysis using Anti-His mouse IgG (Cell Signalling) as shown in Figure S6. The cell pellet was resuspended in PBS containing 6 M urea, lysozyme (0.2 mg/ml) and protease inhibitor cocktail mix (50 µl/ml) and incubated at room temperature for 1 h using a rotational mixer. The cells were sonicated and an additional freeze/thaw step performed to aid in cell lysis. The insoluble cell debris was removed by centrifugation at 38 000 rpm for 90 min. The clarified supernatant was added to 1.5 ml of TALON Metal Affinity Resin (Clontech) and rotated overnight at 4°C using a rotational mixer. The slurry mix was then packed by gravity into a 10 ml Bio-Rad chromatography column. The column was washed twice with PBS, then washed with PBS containing 20 mM imidazole, washed three times with PBS and the bound His-tagged protein was eluted with PBS containing 150 mM imidazole. Residual imidazole was removed from the sample using Econo-Pac 10DG Desalting Columns (Bio-Rad) according to manufacturer's specification. Purity was confirmed by analysis of samples by SDS-PAGE and Western Blot using anti-His antibodies (Bio-Rad). To ensure that the recombinant protein was properly folded, its properties were compared to denatured protein. Denatured protein recombinant Tlp3 was not soluble in PBS or TBS buffers, unlike correctly folded protein. Moreover, the binding affinities of recombinant protein following purification without urea were also tested. No change in binding affinities could be observed (data not shown).

### Identification of ligand binding potential of Tlp3

Amino acid and small molecule arrays and plate based binding assays were performed as previously described. [35]. The ligands investigated by Surface Plasmon Resonance (SPR) included alpha-ketoglutarate, glucosamine, lysine, purine (basic ring), malic acid and L-isoleucine (Sigma). SPR experiments were performed using a Biacore T100 biosensor system (GE- healthcare) at 25°C in 1× PBS pH 7.2 at a flow rate of 30 µl/min. Purified His-Tlp3 was diluted to 0.15 µM in 1× PBS pH 7.2 and loaded on flow cell 2 (FC2) of a Ni^2+^ NTA sensor chip with 5 min contact time. FC1 had no protein loaded and was used as reference. Amino acids were prepared in 1 × PBS pH 7.2 and serially diluted from 0.0125 to 0.2 mM. The amino acids were loaded to the sensor chip using single-cycle kinetics, i.e. after the injection of five amino acid dilutions the chip was regenerated with EDTA. Subsequently, the chip was re-loaded with Ni^2+^ and His-Tlp3 before the injection of the next amino acid to be tested. A 10 min dissociation time was allowed after the addition of each analyte. SPR signals were analyzed using the Biacore Evaluation software to determine K_D_.

### STD NMR spectroscopy

Recombinant Tlp3 (1 mg/mL, 100 µL) dissolved in D_2_O (99.99% D Cambridge Isotopes) containing 50 mM NaCl and 50 mM KH_2_PO_4_ and added to various ligands (1 mg/mL, 150 µL ∼150 mole equivalents) also dissolved in D_2_O (99.99% D Cambridge Isotopes) containing 50 mM NaCl and 50 mM KH_2_PO_4_ to give a total volume of 250 µL in a 3 mm NMR tube for NMR analysis. Control samples were prepared in an identical manner without Tlp3 added. All NMR experiments were performed on a Bruker Avance 600 MHz spectrometer, equipped with a 5-mm TXI probe with triple axis gradients at 283 K without sample spinning. ^1^H NMR spectra were acquired with 32 scans, a 2 s relaxation delay over a spectral width of 6000 Hz. Solvent suppression of the residual HDO peak was achieved by continuous low-power presaturation pulse during the relaxation delay. In the STD-NMR experiments of the chemoattractants (isoleucine, purine and malic acid) and chemorepellents (lysine, arginine and glucosamine) in complex with Tlp3, the protein was saturated at −0.5 ppm in the aliphatic region of the spectrum and off-resonance at 33 ppm with a cascade of 40 selective Gaussian-shaped pulses of 50 ms duration (50 dB), which correlates to a strength of 190 Hz. A 100 µs delay between each soft pulse was applied, resulting in a total saturation time of 2 s and 2 K scans.

Data were obtained with an interspersed acquisition of pseudo-two-dimensional on-resonance and off-resonance spectra in order to minimize the effects of temperature and magnet instability. On- and off-resonance spectra were processed separately, and the final STD-NMR spectrum was obtained by subtracting the individual on- and off-resonance spectra, resulting in less subtraction artefacts. Relative STD effects were calculated according to the equation A_STD_ = (I_0_−I_sat_)/I_0_ = I_STD_/I_0_ by comparing the intensity of the signals in the STD-NMR spectrum (I_STD_) with signal intensities of a reference spectrum (I_0_). The STD signal with the highest intensity was set to 100% and other STD signals were calculated accordingly. A spin lock field of 10 ms was applied to remove unwanted background protein signals. Increased spin lock fields resulted in artefacts and reduced ligand signal intensities. Control STD-NMR experiments were performed using an identical experimental setup and the same ligand concentration but in the absence of the protein.

### Mutagenesis and complementation of *tlp3*

The *tlp3* gene (*cj1564*) in *C. jejuni* 11168-O was inactivated by inverse PCR mutagenesis [69] to produce the strain 11168-OΔ*tlp3*::Km (Table S1). The *tlp3* periplasmic domain (amino acids 43–290) was amplified from the 11168-O genome and cloned into pGEM-TEasy to produce pGU0815 (Table S1). Inverse PCR with primers designed to incorporate a *Bgl*II restriction site and delete 52 bp of the *tlp3* periplasmic domain was followed by insertion of a non-polar kanamycin resistance cassette with a consensus campylobacter promoter from pMW10 [70] in the same orientation as *tlp3* to generate plasmid pGU0817 (Table S1). A kanamycin resistance cassette which lacks the transcription terminator was used in order to minimise effects on genes downstream of *tlp3*. Each construct was verified by DNA sequencing and subsequently electro-transformed into the motile variant of 11168 original clinical isolate, donated by D.G Newell, VIR, London [71]. Replacement of the mutant allele was verified by PCR and DNA sequencing. The complement of this mutant was generated by inserting the complete *tlp3* gene including promoter into the pseudogene *cj0046* using the plasmid pC46 (Table S1). The *tlp3* complement plasmid was electro-transformed into *C. jejuni* 11168-O with insertion into the pseudogene *cj0046* locus to produce strain 11168-OΔ*tlp3*::KmΩ*cj0046*::Cm (Table S1). Complementation was confirmed by PCR and sequence analysis.

### Phenotypic characterisation: Motility, agglutination and biofilm formation

Motility assays were performed as previously described [23] with the following modifications: *C. jejuni* strains were grown microaerobically at 42°C for 24 h in 10 ml Brucella broth (Oxoid) on a shaker (50 rpm/min). Cells were collected by centrifugation at 3000 g for 5 min and washed once with Brucella broth. Equal numbers of bacterial cells (5 µl) of 1 × 10^9^ cfu/ml of each strain of *C. jejuni* were stabbed on top of 0.35% Mueller Hinton Agar plates (MHA Oxoid) and incubated microaerobically at 42°C for 48 h. Autoagglutination (AAG) was performed as previously described [72]. Briefly the cells were harvested with 2 ml of phosphate-buffered saline (PBS pH 7.2) and OD_600 nm_ adjusted to 1.0. The bacterial suspension was poured into sterile glass tubes (13 × 100 mm) and incubated at 25°C, 37°C or 42°C for 24 h. 1.0 ml of the upper aqueous phase was carefully aspirated and the OD_600 nm_ measured. Additionally viable bacteria were enumerated by plate counts. The lower 1 ml of solution containing the majority of the autoagglutinated cells were analysed by scanning electron microscopy (SEM). Biofilm assays were performed according to Reeser *et al* [73] and Tram *et al* [74].

### *In vitro* adherence and invasion in epithelial cell line

Assays were performed as previously described [20] with modifications. Approximately 100 µl of bacterial suspension containing 2 × 10^8^–4 × 10^8^ bacteria per ml was inoculated into 24-well plate containing a confluent monolayer of Caco-2 cells. A 5 min centrifugation step at 500 g was included to facilitate the movement of *C. jejuni* onto the surface of the cell monolayer. The cells were incubated for 45 min at 37°C in a 5% CO_2_ humidified atmosphere to allow passive adherence and internalization. For the adherence assay, the total number of bacteria associated with the cell layer was enumerated by viable count. For the invasion assay, infected cells were washed three times with PBS and 1 ml of MEM (Dibco) containing 400 µg/ml gentamicin was added to the cell monolayer for 3 h. The monolayer was washed three times with PBS and the cells lysed with 200 ml of 0.2% Triton X-100; intracellular bacterial counts were enumerated by viable count. Each assay was performed in triplicate.

### SEM of *C. jejuni*

Cells were grown microaerobically for 24 h at 42°C, collected and washed three times with Brucella broth by centrifugation at 1000 g for 4 min. The final wash step was carried out using PBS and the OD_600 nm_ adjusted to 0.025. Bacteria were fixed on plastic cover slips with 2% glutaraldehyde and 5% formaldehyde solution for 10 min. Slides were washed three times with H_2_O and dehydrated in gradient steps of water/ethanol (15, 30, 50, 75, 90 and 100%). Last steps of dehydration were performed in 50% HMDS/ethanol solution (Hexamethyldisilazane), followed by a final step of 100% HMDS. Slides were left to air dry and subsequently coated with gold (6 nm) prior to analysis on Jeol 5000 Scanning Electron Microscope.

### Chicken colonisation

Chicken colonisation analysis was performed as described previously [39] with an infective dose of 10^8^ CFU (BDD/02/11).

### RNA extraction, cDNA synthesis and RT-qPCR

RNA was extracted using RNeasy kit according to the manufacturer's protocol (Qiagen). cDNA synthesis and RT-qPCR was performed using 11168-O, *Δtlp3* and *Δtlp3*c as previously described [33].

### Modified chemotaxis nutrient depleted agar assay

Nutrient depleted chemotaxis assay was performed as previously described [35] with the following modifications. One or two plugs (6 mm diameter) equidistantly apart (60 mm) were removed from Petri dishes containing 0.5% agar in H_2_O. Each well was filled with 0.5% agar containing 2 mM of selected amino acid. The plates were overlayed with 0.1% agar bacteriological (Oxoid) without nutritional supplements and left for 2 h to allow for diffusion of amino acids to create a chemical gradient. Cultures of *C. jejuni* were adjusted to OD_600 nm_ 1.8 (10^9^ cfu/ml). 100 µl drop of bacterial suspension was inoculated using a micropipette on top of the sloppy agar (0.1% agar bacteriological in H_2_O) in the centre of the Petri dish with the plates incubated at 37°C for 4 h to allow chemotactic migration of the bacteria. To determine the number of viable bacteria associated with each amino acid plug, a 5 mm area around and including each plug was removed and placed into a microcentrifuge tube containing 900 µl of Brucella broth. These were incubated microaerobically for 1 h at 37°C to allow the bacteria to dissociate from the plug into the media. Viable counts were performed with enumeration by serial dilution followed by plate counts. *C. jejuni* 81116 *flaA* ^−^/*flaB* ^−^ isogenic mutant was used as a non-motile, non-chemotactic control; additionally agar plugs containing no amino acid were used as a negative control.

### Yeast two-hybrid and three-hybrid analyses

Yeast two-hybrid and three-hybrid analyses of protein interactions were performed as described previously [35].

## Supporting Information

Figure S1 **Single cycle SPR curves showing concentration dependent interactions between Tlp3 and small molecules.** A, Interaction between Tlp3 and isoleucine; B, Interaction between Tlp3 and lysine; C, Interaction between Tlp3 and glucosamine; D, Interaction between Tlp3 and succinic acid; E, Interaction between Tlp3 and arginine; F, Interaction between Tlp3 and purine; G, Interaction between Tlp3 and malic acid; H, Interaction between Tlp3 and thiamine; I, Interaction between Tlp3 and aspartate; J, Interaction between Tlp3 and α-ketoglutarate; K, No interaction observed between Tlp3 and alanine; L, No interaction observed between Tlp3 and asparagine. All interactions present were tested with a two-fold serial dilution between 12.5 and 200 µM.
(TIF)

Figure S2 **Comparison of autoagglutination in** ***C. jejuni*** **strains.** Image of autoagglutination in static suspension after 24 h in strains (1) 11168-O, (2) Δ*tlp3*, (3) Δ*tlp3*c, (4) 81116Δ*flaA*/*flaB* and (5) 81–176.
(TIF)

Figure S3 **Avian colonisation of chicken caeca by** ***C. jejuni*** **.** Δ*tlp3* shows no defect for chick colonisation. Each point represents the CFU/g caecal content of an individual chick 5 days post infection.
(TIF)

Figure S4 **SEM of Δ** **tlp3 aggregating on the surface of chicken caeca** **.** Presence of *C. jejuni* 11168-O (S4A) and Δ*tlp3* (S4B) cells in avian caeca were assessed by dissecting a portion of the chicken caeca and visualising it under SEM, 5 days post infection. SEM images of *C. jejuni* 11168-O Δ*tlp3* illustrate highly aggregated formation of cells on the surface and in the crevices of chicken caeca, and presence of extracellular polysaccharide and loosely associated cells for *C. jejuni* 11168-O WT cells. Scale: 2–5 µm.
(TIF)

Figure S5 **Amino acid sequence alignment of the MCP signalling domains of** ***C. jejuni*** **group A Tlps 1, 2, 3 and 4.** Underlined residues represent the region homologous to the shortest fragment of the *E. coli* serine chemoreceptor, Tsr (residues 350–471), required for CW-signalling and stimulation of CheA histidine kinase activity and are, therefore, involved in binding CheW [41]. Alignment was performed using CLUSTALW 2.1.
(TIF)

Figure S6 **Coomassie blue stained PAGE and corresponding Western blot of expressed and purified Tlp3.** A, Lane 1 and 6: Bio-Rad Precision Plus Protein™ All Blue Standards. Lanes 2 and 4: Coomassie blue stained protein from uninduced *E. coli* BL21 DE3Gold containing the Tlp3 expression vector. Lanes 3 and 5: Coomassie blue stained protein from *E. coli* BL21 DE3Gold containing the Tlp3 expression vector induced with IPTG. Lanes 7 and 9: Western blot of the uninduced samples. Lanes 8 and 10: Western blot of the induced samples; B, Lane 1: Bio-Rad Precision Plus Protein™ All Blue Standards. Lane 2: Flow through of purification process. Lane 3–10: Eluted fractions of purified Tlp3.
(TIF)

Movie S1 **Assessment of motility in fluorescently labelled** ***C. jejuni*** **11168-O, Δ** ***tlp3*** **and Δ** ***tlp3*** **c.** Live imaging of culture grown *C. jejuni* was performed in a heated culture chamber set to 37°C using a Nikon Eclipse Ti microscope. Bacteria were grown and prepared as described in materials and methods. *C. jejuni* 11168-O, Δ*tlp3* and Δ*tlp3c* cells were fluorescently labelled with FITC (Fluorescein-5-EX, Succinimidyl Ester, Molecular Probes) in Brucella broth and visualised on the BSA blocked glass-bottom dish at 60×. Movements were captured for 15–20 s, with video footage showing normal run and tumble bias for *C. jejuni* 11168-O (Movie S1A), inhibition of smooth swimming with enhanced rate of tumbles by the Δ*tlp3* (Movie S1B) and the restored run and tumble movement by the complemented mutant Δ*tlp3c* (Movie S1C).
(ZIP)

Table S1 **Microbial strains and plasmids used in this study.** A summary of the microbial strains, mutants and plasmid used throughout this study.
(DOCX)

Table S2 **List of primers used in this study.** A summary of primers used throughout this study.
(DOCX)
