# Supplementary figures and images for: Characterisation of a Multi-ligand Binding Chemoreceptor CcmL (Tlp3) of Campylobacter jejuni
Source: PLoS Pathog. 2014 Jan 2;10(1):e1003822. doi: 10.1371/journal.ppat.1003822 (PMC3879368; doi:10.1371/journal.ppat.1003822)

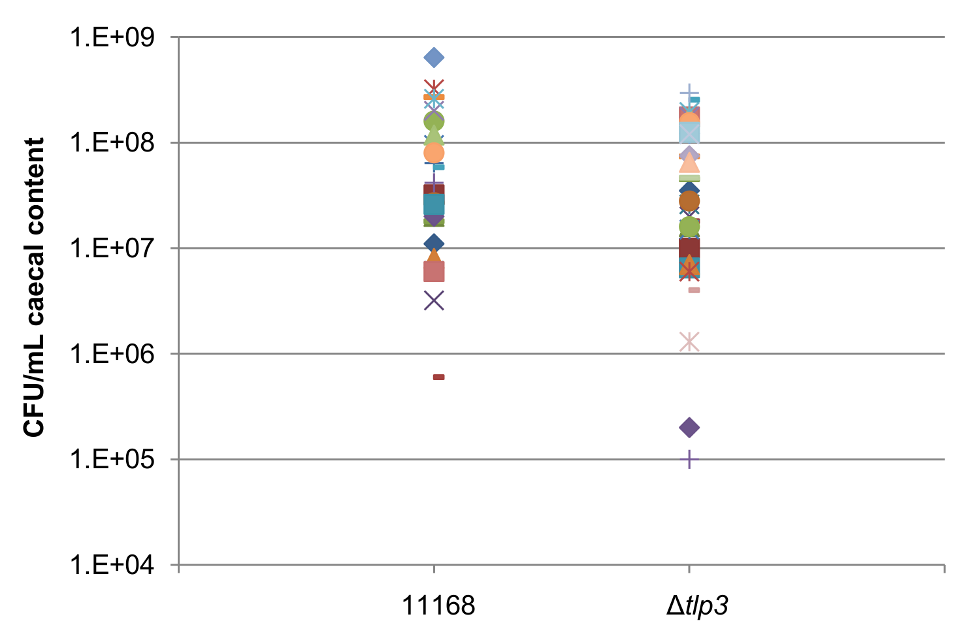

Supplement: Figure S3 — Avian colonisation of chicken caeca by C. jejuni . Δtlp3 shows no defect for chick colonisation. Each point represents the CFU/g caecal content of an individual chick 5 days post infection. (TIF) [file ppat.1003822.s003.tif]

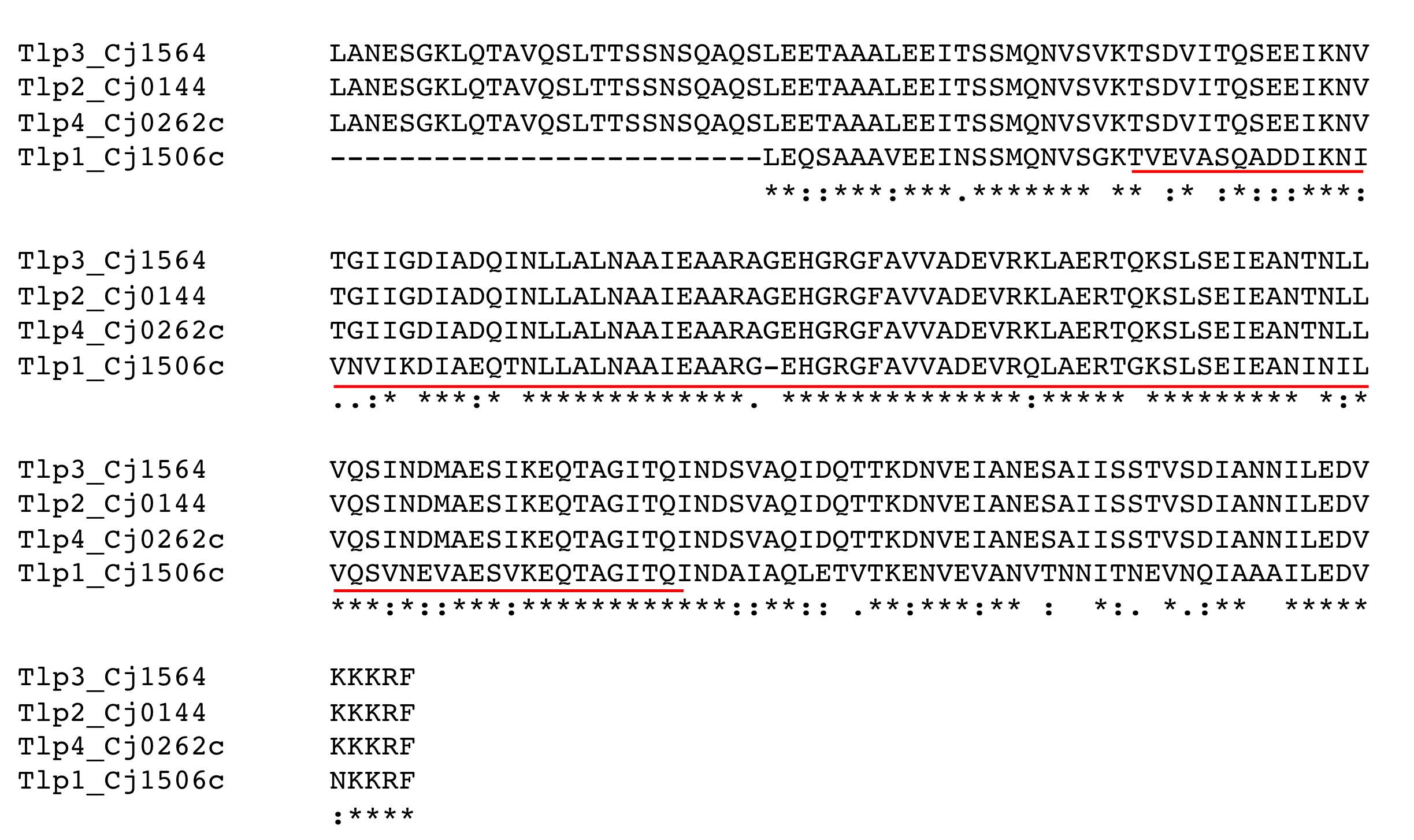

Supplement: Figure S5 — Amino acid sequence alignment of the MCP signalling domains of C. jejuni group A Tlps 1, 2, 3 and 4. Underlined residues represent the region homologous to the shortest fragment of the E. coli serine chemoreceptor, Tsr (residues 350–471), required for CW-signalling and stimulation of CheA histidine kinase activity and are, therefore, involved in binding CheW [41]. Alignment was performed using CLUSTALW 2.1. (TIF) [file ppat.1003822.s005.tif]
