# Supplementary material for: Characterisation of a Multi-ligand Binding Chemoreceptor CcmL (Tlp3) of Campylobacter jejuni
Source: PLoS Pathog. 2014 Jan 2;10(1):e1003822. doi: 10.1371/journal.ppat.1003822 (PMC3879368; doi:10.1371/journal.ppat.1003822)
Supplement: Table S1 — Microbial strains and plasmids used in this study. A summary of the microbial strains, mutants and plasmid used throughout this study. (DOCX) [file ppat.1003822.s008.docx]

**Table S1.** Microbial strains and plasmids used in this study.

| **Bacterial strains/ plasmids** | **Description** | **Reference/source** |
| --- | --- | --- |
| *C. jejuni* 11168-O | Wild type (human isolated strain) originally strain 5636/77 | (Skirrow, 1977) |
| *C. jejuni*11168-O*∆tlp3::aphA3* | Isogenic mutant of *tlp3* | This study |
| *C. jejuni*11168-OΔ*tlp3 ::aphA3 Ωcj0046::cat* | Complemented *tlp3^-/+^* mutant in *C. jejuni* 11168-OΔ*tlp3::Km* | This study |
| *C. jejuni* 81116Δ*flaA^-^/flaB^-^* | Isogenic mutant of *flaA/flaB* | J.M. Ketley |
| 11168-O*∆cheV::cat* | Isogenic mutant of *cheV* | [1] |
| 11168-O*∆cheW::cat* | Isogenic mutant of *cheW* | [1] |
| *E. coli* DH5α | F^–^, ø80d*lacZ*ΔM15, Δ(*lacZYA-argF*)U169, *deo*R, *recA*1, *endA*1, *hsdR*17(rK^–^, mK^+^), *phoA*, *supE*44, λ–, *thi*-1, *gyrA*96, *relA*1 | [2] |
| *E. coli* BL21(DE3) | F^-^ *ompT hsdS_B_* (r_B_^-^ m_B_^-^) *gal dcm* (DE3) | Novagen |
| pGEM^®^-T Easy | Intermediate cloning vector | Promega |
| pGU0509 | Km^R^ with promoter cloned into pGEM^®^-T Easy | [3] |
| pGU0815 | *tlp3^peri^* cloned into pGEM^®^-T Easy | This study |
| pET-19b | His-tag expression vector | Novagen |
| pGU0816 | *tlp3^peri^* cloned into pET-19b | This study |
| pGU0817 | *∆tlp3*::Km^R^ cloned into pGEM^®^-T Easy | This study |
| pAV35 | Chloramphenicol resistant | [4] |
| pRRK | *C. jejuni* rRNA spacer integration vector; Km^R^ | J.M Ketley |
| pC46 | *C. jejuni* *cj0046* pseudogene integration vector; Cm^R^ | [5] |
| **Yeast strains** |  |  |
| *Saccharomyces cerevisae* AH109 | MATa, trp1-901, leu2-3, 112, ura3-52, his3-200, gal4Δ, gal80Δ, LYS2:GAL1_UAS_-GAL1_TATA_-HIS3, GAL2_UAS_-GAL2_TATA_-ADE2, URA3:MEL1_UAS_-MEL1_TATA_-lacZ | Clontech |
| **Yeast two-hybrid plasmids** |  |  |
| pGADT7 | GAL-4 activation domain expression vector | Clontech |
| pGBKT7 | GAL-4 DNA binding domain expression vector | Clontech |
| pGADT7-T | Allows expression of the SV40 large T-antigen as a fusion to the GAL-4 AD | Clontech |
| pGBKT7-53 | Allows expression of the murine p53 protein as a fusion to the GAL-4 DNA-BD, used as positive control when co- transformed with pGADT7-T | Clontech |
| pGBKT7-Lam | Allows expression of the human lamin C protein as a fusion to the GAL-4 DNA-BD, used as a negative control when co-transformed with pGADT7-T | Clontech |
| pADcheW | *cheW* cloned into pGADT7, Amp^R^ | [1] |
| pADcheA | *cheA* cloned into pGADT7, Amp^R^ | [1] |
| pADcheA^dHK^ | DNA sequence encoding the histidine kinase domain of *cheA*, Amp^R^ | [1] |
| pADcheA^dRR^ | DNA sequence encoding the response regulator domain of *cheA* cloned into pGADT7, Amp^R^ | [1] |
| pADcheY | *cheY* cloned into pGADT7, Amp^R^ | [1] |
| pADcheV | *cheV* cloned into pGADT7, Amp^R^ | [1] |
| pADcheV^dW^ | DNA sequence encoding the CheW-like domain of CheV cloned into pGADT7, Amp^R^ | [1] |
| pADcheV^dRR^ | DNA sequence encoding the response regulator domain of CheV cloned into pGADT7, Amp^R^ | [1] |
| pADcheB | *cheB* cloned into pGADT7, Amp^R^ | [1] |
| pADtlp234^sig^ | DNA sequence encoding residues 513 – 659 of Tlp2 (Cj0144), residues 517 – 662 of Tl3 (Cj1564) and residues 520 – 665 of Tlp 4 (Cj0262c) cloned into pGADT7, Amp^R^ | This study |
| pBKcheW | *cheW* cloned into pGBKT7, Km^R^ | [1] |
| pBKcheA | *cheA* cloned into pGBKT7, Km^R^ | [1] |
| pBKcheA^dHK^ | DNA sequence encoding the histidine kinase domain of *cheA* cloned into pGBKT7, Km^R^ | [1] |
| pBKcheA^dRR^ | DNA sequence encoding the response regulator domain of *cheA* cloned into pGBKT7, Km^R^ | [1] |
| pBKcheY | *cheY* cloned into pGBKT7, Km^R^ | [1] |
| pBKcheV | *cheV* cloned into pGBKT7, Km^R^ | [1] |
| pBKcheV^dW^ | DNA sequence encoding the CheW-like domain of CheV cloned into pGBKT7, Km^R^ | [1] |
| pBKcheV^dRR^ | DNA sequence encoding the response regulator domain of CheV cloned into pGBKT7, Km^R^ | [1] |
| pBKcheB | *cheB* cloned into pGBKT7, Km^R^ | [1] |
| pBKtlp234^sig^ | DNA sequence encoding residues 513 – 659 of Tlp 2 (Cj0144), residues 517 – 662 of Tlp 3 (Cj1564) and residues 520 – 665 of Tlp 4 (Cj0262c) cloned into pGBKT7, Km^R^ | This study |
| **Yeast three-hybrid plasmids** |  |  |
| pBridge | Contains 2 multiple cloning sites to allow expression of one protein as a fusion to the GAL-4 DNA-BD and a second protein expressed under the control of the conditional MET25 promoter, Amp^R^ | Clontech |
| pBrWIVII | pBridge – *cheW* cloned into MCSI  *cheV* cloned into MCSII, Amp^R^ | [1] |
| pBrVIWII | pBridge – *cheV* cloned into MCSI  *cheW* cloned into MCSII, Amp^R^ | [1] |
| pBrTlp234^sig^IWII | pBridge – *tlp234^sig^* cloned into MCSI  *cheW* cloned into MCSII, Amp^R^ | This study |
| pBrTlp234^sig^IVII | pBridge – *tlp234^sig^* cloned into MCSI  *cheV* cloned into MCSII, Amp^R^ | This study |

**References**

1. Hartley-Tassell LE, Shewell LK, Day CJ, Wilson JC, Sandhu R, et al. (2010) Identification and characterization of the aspartate chemosensory receptor of *Campylobacter jejuni*. Mol Microbiol 75: 710-730.

2. Hanahan D (1983) Studies on transformation of *Escherichia coli* with plasmids. J Mol Biol 166: 557-580.

3. Klipic Z (2011) Characterisation of *Campylobacter jejuni* glycoprotease and its role in bacteria - host interactions. Griffith University. Institute for Glycomics.

4. Van Vliet AH, Wood A, Henderson J, Wooldridge KG, Ketley J (1998) Bacterial pathogenesis. In: Williams P, Ketley JM, Salmond G, editors. Techniques for bacterial pathogenesis. San Diego: Academic Press. pp. xix, 620 p.

5. Gaskin DJH, Van Vliet AHM, Pearson BM (2007) The *Campylobacter* genetic toolbox: development of tractable and generally applicable genetic techniques for *Campylobacter jejuni*. Zoonoses and Public Health 54: 101-101.
