# Supplementary material for: Characterisation of a Multi-ligand Binding Chemoreceptor CcmL (Tlp3) of Campylobacter jejuni
Source: PLoS Pathog. 2014 Jan 2;10(1):e1003822. doi: 10.1371/journal.ppat.1003822 (PMC3879368; doi:10.1371/journal.ppat.1003822)
Supplement: Table S2 — List of primers used in this study. A summary of primers used throughout this study. (DOCX) [file ppat.1003822.s009.docx]

**Table S2.** List of primers used in this study.

| **Primer name** | **Sequence 5’-3’** | **Restriction Site** | **Reference** |
| --- | --- | --- | --- |
| TLP3F | CATATGAAAACCTCACTATATGAAAGCACTCTTA | *Nde*I | This study |
| TLP3R | CTCGAGTTATGCAGCTTTATAAATAGGTTTAATTTATAATATC | *Xho*I | This study |
| TLP3MUTF | AGATCTTAACGTATTCTAAAGCTATTTATAAAAT | *Bgl*II | This study |
| TLP3MUTR | CATTTGTTTTTAAAGCTTCTTGATACCAAT. |  | This study |
| T3FL F | ATCGTCTCACATGCTAAAAATAACAAAGATTAAAAG | *Bsm*BI | This study |
| T3FL R | ATCGTCTCACTTAAAACCTCTTCTTCTTAACATC | *Bsm*BI | This study |
| T3Comp | CACCAGCATATTTAGATACAGTTTTAAAAC |  | This study |
| CAT F NW | GAAGATCTGATTGAAAAGTGGATAGATTTATG | *Bgl*II | This study |
| CAT R NW | GAAGATCTATTTTTCGATTGGCGCGCCTGAGGG | *Bgl*II | This study |
| pC46 Cat F | ATGATGCAATTCACAAAGATT |  | This study |
| pC46 CatR | TTATTTATTCAGCAAGTCTTG |  | This study |
| Topo Kan F | ATGATTGAACAAGATGGATTGC |  | This study |
| Topo Kan R | TCAGAAGAACTCGTCAAGAAGG |  | This study |
| pK46 Kan F | AATGGCTAAAATGAGAATATC |  | This study |
| pK46 Kan R | AAACAATTCATCCAGTAAAAT |  | This study |
| 5’AD | CTATTCGATGATGAAGATACCCCACCAAAC |  | Clontech |
| 3’AD | GTGAACTTGCGGGGTTTTTCAGTATCTACG |  | Clontech |
| 5’DNA-BD | TCATCGGAAGAGAGTAGT |  | (Clontech) |
| 3’DNA-BD | GTCACTTTAAAATTTGTATAC |  | (Clontech) |
| 5’MCSII | ATCCTTATGACGTGCCTGAC |  | (Clontech) |
| 3’MCSII | GAAAGCAACACCTGGCAATTC |  | (Clontech) |
| tlp234^sig^EcoRIF | GAATTCATGCAAAATGTTTCAGTTAAA | *Eco*RI | This study |
| tlp234^sig^BamHIR | GGATCCAAACCTCTTCTTCTTAACATC | *Bam*HI | This study |
| gyrA-QRT-F | ATGCTCTTTGCAGTAACCAAAAAA |  | [1] |
| gyrA-QRT-R | GGCCGATTTCACGCACTTTA |  | [1] |
| pgp1-QRT-F | AACCGTGCAACGCATTAAAGA |  | [1] |
| pgp1-QRT-R | AAAACCCCAACCATCGTGAA |  | [1] |
| Therm 1 | TATTCCAATACCAACATTAGT |  | [2] |
| Therm 2.1 | GAAGATACGGTGCTATTTTG |  | [2] |
| cj1563-QRT-F | TGGATAGAATGGCTTAGAATTTCAGGT |  | This study |
| cj1563-QRT-R | CTTTGTTTGCTTTAACATCTCTTGTC |  | This study |
| pflA-QRT-F | AATGCTATCAATAGATATCGTGGCAG |  | This study |
| pflA-QRT-R | ATCACGCATACTTGGATCTTGAGT |  | This study |
| flaA-QRT-F | GAGCAGGAGCTACTTCAGATACTTTTG |  | This study |
| flaA-QRT-R | ATTGCAGCAACTAAGGCTCCATTAGC |  | This study |

**References**

1. Frirdich E, Biboy J, Adams C, Lee J, Ellermeier J, et al. (2012) Peptidoglycan-modifying enzyme Pgp1 is required for helical cell shape and pathogenicity traits in *Campylobacter jejuni*. PLoS Pathog 8: e1002602.

2. Hartley-Tassell LE, Shewell LK, Day CJ, Wilson JC, Sandhu R, et al. (2010) Identification and characterization of the aspartate chemosensory receptor of *Campylobacter jejuni*. Mol Microbiol 75: 710-730.
